# Prevalence of bovine tuberculosis and distribution of tuberculous lesions in cattle slaughtered at Gondar, Northwest Ethiopia

**DOI:** 10.1080/20008686.2021.1986919

**Published:** 2021-11-01

**Authors:** Abebe Belete, Selam Tilahun, Belete Haile, Yitayew Demessie, Seleshe Nigatu, Abebaw Getachew, Gashaw Getaneh, Elias Kebede, Mebrat Ejo

**Affiliations:** a Department of Veterinary Epidemiology and Public Health, College of Veterinary Medicine and Animal Sciences, University of Gondar, Gondar, Ethiopia; b Department of Biomedical Sciences, College of Veterinary Medicine and Animal Sciences, University of Gondar, Gondar, Ethiopia; c Department of Veterinary Pharmacy, College of Veterinary Medicine and Animal Sciences, University of Gondar, Gondar, Ethiopia

**Keywords:** Acid-fast bacilli, cattle, histopathology, microscopy, post-mortem inspection, tuberculosis

## Abstract

**Background:**

In Ethiopia, bovine tuberculosis (BTB) is a neglected disease that affects the economy and livelihoods of farmers. However, the available data is limited due to insufficient disease surveillance in the country. Therefore; this study aimed to assess the prevalence and distribution of lesions of BTB in cattle slaughtered at Gondar, Northwest Ethiopia.

**Methods:**

Postmortem examinations were used to detect tuberculous lesions, while smear microscopy and histopathology were performed for the identification of acid-fast bacilli (AFB).

**Results:**

Of 497 inspected slaughtered cattle, 45 (9.1%, 95%CI; 0.0668–0.1193) were diagnosed with BTB suggestive tuberculous lesions. A higher proportion of gross lesions was recorded in lymph nodes of lungs; at the mediastinal (14, 31.1%) and bronchial (10, 22.2%) lymph nodes, and followed by mesenteric lymph nodes (9, 20%). Of 45 tuberculous lesions; only 2 (4.4%) were identified as AFB positive by smear microscopy and histopathology. In the overall statistical analysis, body conditions of slaughtered cattle were found to be significantly associated with BTB tuberculous lesions (p < 0.05).

**Conclusion:**

This finding provides the prevalence of BTB and distribution of tuberculous lesions in cattle slaughtered at the abattoir and highlights the need for a practicable control strategy of the disease in the region.

## Introduction

Bovine tuberculosis (BTB), caused by *Mycobacterium bovis* (*M. bovis*), is a chronic infectious bacterial disease affecting domestic animals [1,2] and wildlife population [3,4], with a risk of zoonosis in humans [5–7]. Although cattle are considered the primary hosts, BTB has one of the broadest host ranges among infectious diseases [2,8]. The disease is usually characterized by the formation of granulomas in the tissues and organs, more significantly in the lungs, lymph nodes, intestine, kidney, and others [2,9].

In many developing countries with limited resources, including Ethiopia, BTB is one of the most important public health problems and is reported as re-emerging zoonosis [6,10,11], because of shortages in disease prevention and control measures, including lack of regular milk pasteurization and slaughterhouse meat inspection [5,12]. In Ethiopia, few studies have been reported *M. bovis* infection in humans [13–15] and suggested the occurrence of animal-to-human transmission. This situation is aggravated by the presence of various risk factors such as human consumption habits of raw or undercooked meat and unpasteurized dairy products [13,16,17], and the high prevalence of HIV infections [5,6,12,18]. In the country, consuming raw meat is a welcome tradition; thus, meat may also remain to be another area of concern or threat to be a source of BTB infection in humans. Studies have also estimated that *M. bovis* accounts for 37.7% of human tuberculosis (TB) in Africa [6], and, in Ethiopia, 0.7% to 17% of *M. bovis* has been isolated from lymph node aspirates of TB patients [19,20]. Besides, the disease causes substantial animal health-induced economic losses due to the reduced production of affected animals and the elimination of affected animal carcasses at slaughter, and animal trade restrictions [2,21].

In Ethiopia, various studies in different areas have reported the prevalence of BTB; it ranges from 3.5% to 5.2% in slaughterhouses and 3.5–50.0% in dairy farms [22–25]. The disease negatively affects the economy and the livelihoods of farmers, particularly in poor and marginalized communities [16,26–28]. However, the available data is limited due to insufficient disease surveillance and the lack of better diagnostic facilities in the country. Abattoir inspection remains an economically affordable and valuable technique to detect BTB in carcasses of slaughtered animals at slaughterhouses in Ethiopia [24,29–31], and also a very important component for the investigation of the disease [9,32]. Moreover, the recognition of gross lesions of BTB in cattle has supported the surveillance and test and slaughter control programs of the disease from an animal population in areas with low and high prevalence [2,9]. The BTB eradication program continues to depend on slaughter surveillance as the most economically efficient method of detecting cattle infected with *M. bovis* [9].

Despite the significant economic impacts and zoonotic risks associated with BTB [7,33], the available data of BTB prevalence are scarce in many areas of Ethiopia. In addition, in the country, most communities of smallholders and animal keepers share the same houses and air spaces with animals, and have insufficient knowledge on risks involved through consumption of animal products, thereby risking contracting zoonotic infections [16,34]. In particular, studies on the prevalence of BTB in cattle slaughtered in an abattoir in the Amhara region are inadequate. Such information is critical to monitor the transmission and spread of the disease among cattle and also for appropriate control strategies with herd testing and postmortem inspection at the slaughterhouse in the region as well as the country. Therefore; this study was aimed to investigate the prevalence of BTB and assess the distribution of tuberculous lesions among tissues and/or organs of slaughtered cattle at Gondar, Northwest Ethiopia.

## Materials and methods

### Study location and abattoir

**Figure 1.**
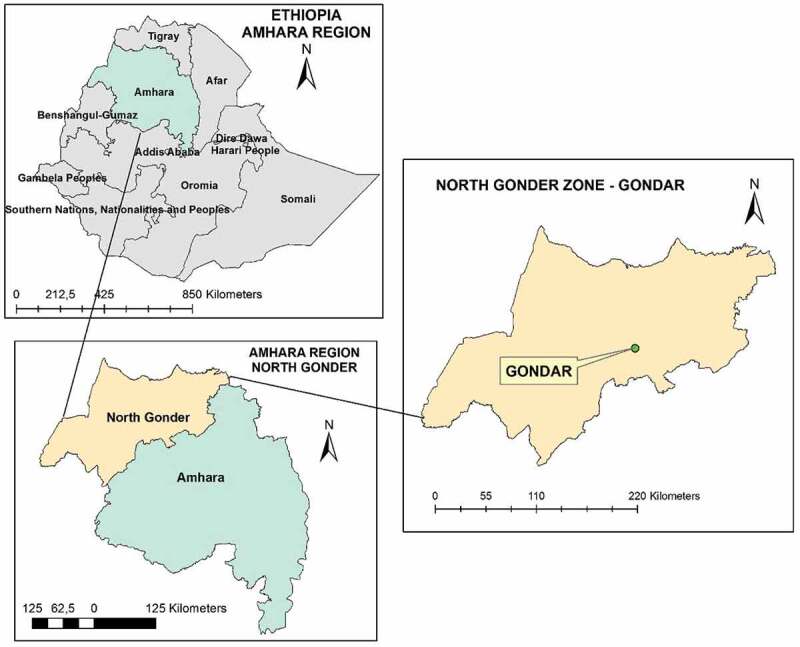
Map of the study area

The study was conducted at ELFORA abattoir, in Gondar town, which is located 740 km far from the capital city, Addis Ababa, in the Northwest of Ethiopia at 12° 36ʹ 10.8648” N and 37° 27ʹ 7.6752” E, (Figure 1). The current study area, North Gondar, is also known for its livestock population, livestock markets, and trade routes between the neighboring regions and bordering Sudan [35]. The abattoir was selected based on the high number of cattle slaughtered annually and the wide topographical origin of the animals. It is also the biggest slaughterhouse and delivers animal slaughter services for Gondar town and the surrounding community; butcher houses, supermarkets, hotels, and restaurants. It receives cattle from various parts of the Amhara region, including Gondar town and surrounding livestock-rich districts such as Dembia, Wogera, Maksegnit, Metema, and Aymba [35]. Livestock is the vital property in this study area; involving more than 3.2 million cattle, 1.3 million sheep, and 1.8 goats [35].

### Study design and animals

A cross-sectional study with a systematic random sampling method was conducted between October 2017 and June 2018. The selection of cattle to be included in the study was made arbitrarily among cattle slaughtered each day. For this study, among cattle slaughtered each day; on average 7 heads of cattle, were considered for post-mortem examination with a proper undertaking of detail necropsy inspection. Slaughter cattle examination was performed 5 days per week except for Christian fasting events, including Wednesday and Friday when animals were not slaughtered due to fasting days.

All cattle that were presented for slaughter were local zebu breed, and the sex of examined animals was male, while female animals were not slaughtered in an abattoir during this study. Besides, animal identification and animal-related variables such as age and body condition score (BCS) of the individual animal were recorded. The age of cattle was determined based on dental eruption as described previously [36], and categorized as an adult (if ≤ 7 years old) and old ages (if >7 years old). Additionally, BCS was made using a method developed for Zebu cattle [37], and the observation of anatomical parts such as vertebral column, ribs, and spines. The study animals were grouped as poor [1], medium [2 and 3], or good [4 and greater] body condition.

### Sample size

The sample size was calculated assuming the expected prevalence of BTB lesions to be 11.6% [38], using a 95% confidence interval (CI) and 5% absolute precision based on the formula described by Thrusfield [39].

$$n=\frac{1.96^{2}X\;P_{exp}\left(1-P_{exp}\right)}{d^{2}}$$

Where n = required sample size; P_exp_ = expected prevalence; d^2^ = desired absolute precision (usually 0.05)

Subsequently, a total of 497 slaughtered cattle were examined for tuberculous lesions suspected of BTB, for proportionate sampling.

### Antemortem and postmortem examinations

Clinical examination of cattle was conducted physically following the antemortem examination protocol [40] before they were slaughtered. Specifically, examination of superficial lymph nodes, visible mucus membrane, and body conditions was done for the individual animal.

Postmortem examinations for BTB were carried out according to the procedure described by the meat inspection manual for developing countries [41]. Additionally, detailed post-mortem examinations (inspection, palpation, and incision) of the carcass, lungs, liver, kidneys, and intestines together with the lymph nodes were performed as recommended by Corner *et al*. [42,43]. Lymph nodes, such as retropharyngeal, mandibular, mediastinal, bronchial, hepatic, and mesenteric lymph nodes, were carefully inspected, palpated, and incised into small size sections and then be visualized under bright light for detection of gross BTB suggestive lesions [42,43]. Inspected cattle were categorized as lesioned when BTB suggestive lesions are found in any of the tissues or organs examined, and non-lesioned when no lesion was found in any of the tissues or organs inspected.

### Sample collection and transportation

Suspected macroscopic tuberculous lesions from slaughtered cattle were collected under aseptic conditions in a screw-capped universal bottle containing 5 ml of sterile 0.85% saline water, and then transported by maintaining a cold chain to Pathology laboratory, University of Gondar Comprehensive Specialized Hospital, and kept at −20°C until the acid-fast stain and histopathology analysis.

### Identification of Acid-fast bacilli (AFB)

#### Smear microscopy

Preliminary identification of tubercle bacilli was done primarily by observation of acid-fast bacilli (AFB) using Ziehl–Neelsen (ZN) smear microscopy following the World Health Organization (WHO) protocol [44]. Briefly, frozen samples were thawed overnight before processing, removed from containers, and put into sterile Petri dishes. Then, the tissue samples were sectioned into pieces using a sterile scalpel blade and homogenized manually by pestle and mortar in 10 ml of sterile normal saline solution for 10 min. Next, homogenates were decontaminated with 2 ml of 4% NaOH for 15 min, neutralized with 1% (0.1 N) hydrochloric acid (HCl) using phenol red as an indicator, and concentrated by centrifugation at 3000 x g for 15 min. The supernatant was discarded, and an aliquot of the pellet from each processed sample was used to prepare a smear for ZN. The pellet was gently smeared on a clean, glass microscopic slide, air-dried, and heat-fixed. The stained slides were then observed under a 100X objective of a light microscope to determine the presence and morphology of AFB [44]. Simultaneously, negative and positive control smears were used to assess the quality of the staining procedures.

#### Histopathology

Collected tissue and organ samples were fixed with 10% formalin and then processed by the paraffin embedding technique [45]. Samples possessing fat and heavy calcification were trimmed and decalcified before paraffin embedding. Briefly; a section from each piece of tissues and organs with gross lesions was embedded in standard paraffin wax (Shandon Hypercenter XP), and sectioned at 5 μm thickness by using a microtome. Then slides with sections were prepared and stained with Haematoxylin-Eosin (H&E) method. Stained slides were evaluated microscopically at increasing magnifications. A sample was considered positive if lesions characteristic for BTB had shown granulomatous inflammation with central caseous necrosis or calcifications [45].

### Statistical analysis

The collected data from the abattoir and laboratory were entered in a Microsoft Excel spreadsheet and analyzed using SPSS version 20 (IBM SPSS Statistics, USA). Descriptive statistics were used to analyze the overall proportion of tuberculous lesions and lesion frequency in different anatomical sites. Pearson’s chi-square test (χ2) was performed to determine the association of age and body conditions of cattle with the occurrence of BTB suggestive lesions and prevalence of BTB. A p-value of <0.05 was considered statistically significant.

## Results

### Slaughter cattle characteristics

A total of 497 slaughtered cattle were examined in this study; of which 425 were from adults and 72 from the old age category. All slaughtered cattle were male and local zebu breed, with the majority of the cattle (85.5%) categorized in the adult age category (≤ 7 years old). The BCS of slaughtered cattle displayed 141 (28.4%) good, 176 (35.4%) medium, and 180 (36.2%) poor body condition, (Table 1).

**Table 1.** Association of animal risk factors with tuberculous lesions in cattle slaughtered at Gondar ELFORA abattoir

Variable	No. of cattle examined (n = 497) (%)	Positive for TB-lesion (n = 45) (%)	χ2	p-value
**Age category**			0.046	0.831
adult	425 (85.5)	38 (84.4)		
old	72 (14.5)	7 (15.6)		
**Sex**			-	-
male	497 (100)	45 (100)		
**Breed**			-	-
local	497 (100)	45 (100)		
**Body condition score (BCS)**			8.135	**0.017**
good	141 (28.4)	8 (17.8)		
medium	176 (35.4)	12 (26.7)		
poor	180 (36.2)	25 (55.5)		

χ2: Pearson’s chi-square test (χ2); BCS: Body Condition Scoring; Statistically significant if p-value <0.05.

### The overall prevalence of BTB

Among the total 497 inspected slaughtered heads of cattle, 45 (9.1%, 95%CI; 0.0668–0.1193) were found with BTB suggestive tuberculous lesions. The high frequency of tuberculous lesions was observed in the age category of adult (<7 years old) (38, 84.4%), and poor body conditioned slaughtered cattle (25, 55.5%), (Table 1). In the overall statistical analysis, body conditions of slaughtered cattle were found to be significantly associated with BTB tuberculous lesions (p < 0.05), while age category did not have any variation (Table 1).

### Distribution of tuberculous lesions from tissues and organs

Of the total 45 BTB suggestive lesions detected during the postmortem examination; a higher proportion was identified in the lymph nodes of the lungs; specifically at the mediastinal (14, 31.1%) and bronchial (10, 22.2%) lymph nodes, and followed by the mesenteric lymph nodes (9, 20%) and lymph nodes of the head (mandibular (4, 8.9%) and retropharyngeal (4, 8.9%) lymph nodes). The least distribution of BTB suggestive tuberculous lesions was observed from lungs tissue (2, 4.4%), and hepatic lymph node (2, 4.4%), (Table 2). Only a single lesion in any of the tissues or organs examined per one slaughtered cattle had been taken as BTB suggestive lesions.

**Table 2.** Distribution and occurrence of BTB suspected tuberculous lesions among slaughtered cattle at Gondar ELFORA abattoir, Ethiopia

Anatomical sites	Distribution of BTB lesions (n = 45) (%)	Ziehl-Neelson staining		Histopathology	
		Positive (n = 2) (%)	Negative (n = 43) (%)	Positive (n = 2) (%)	Negative (n = 43) (%)
Retropharyngeal	4 (8.9)	0 (0.0)	4 (9.3)	0 (0.0)	4 (9.3)
Mandibular	4 (8.9)	0 (0.0)	4 (9.3)	0 (0.0)	4 (9.3)
Bronchial	10 (22.2)	1 [50]	9 (20.9)	1 [50]	9 (20.9)
Mediastinal	14 (31.1)	1 [50]	13 (30.2)	1 [50]	13 (30.2)
Mesenteric	9 [20]	0 (0.0)	9 (20.9)	0 (0.0	9 (20.9)
Hepatic	2 (4.4)	0 (0.0)	2 (4.7)	0 (0.0)	2 (4.7)
Lungs	2 (4.4)	0 (0.0)	2 (4.7)	0 (0.0)	2 (4.7)

**Figure 2.**
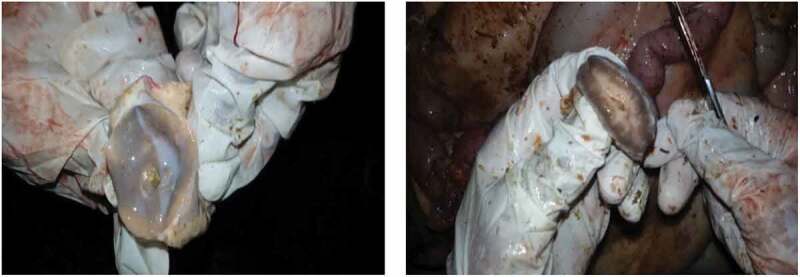
BTB suggestive gross lesions on retropharyngeal (left) and mediastinal (right) lymph nodes among slaughtered cattle

Macroscopically, the most common change seen in BTB suspected tuberculous lesioned tissues and organs was the presence of circumscribed yellowish-white lesions of various sizes. The lesioned tissues were dry and gritty with coalescing areas of caseous and calcified necrosis (Figure 2).

### Acid-fast bacilli (AFB) identification

**Figure 3.**
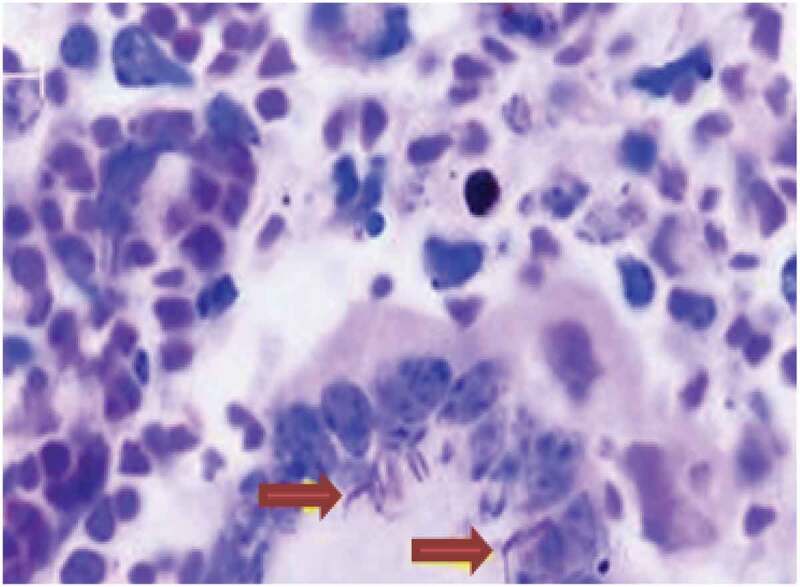
A granulomatous lesion with ZN staining: indicates the presence of giant cells with AFB positive tuberculous bacilli (arrows)

In this study, out of 45 BTB suspected tuberculous lesions, only 2 (4.4%) were identified as acid-fast bacilli (AFB) by smear microscopy (Ziehl–Neelsen) and morphological characteristics, (Table 2), while the remained 43 (95.6%) were AFB smear-negative. Both AFB smear-positive tuberculous samples were from lymph nodes collected in two different slaughter cattle, one from bronchial and the other from mediastinal lymph nodes; with scanty AFB detected per slide, (Figure 3).

**Figure 4.**
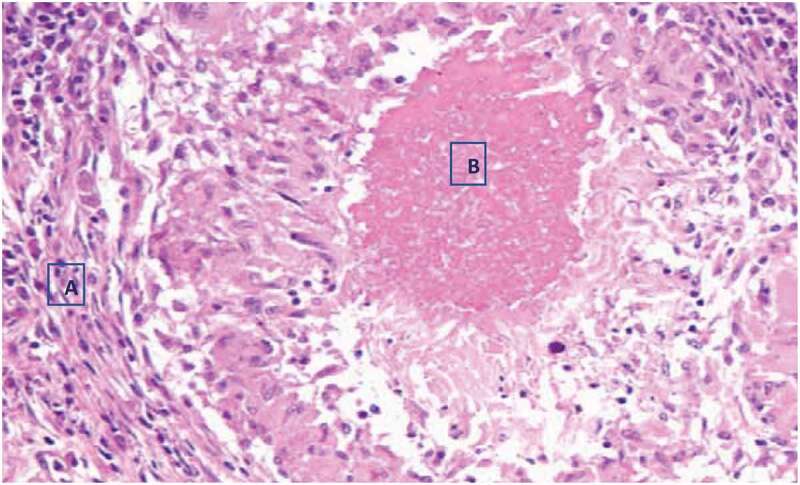
Histopathology of BTB suspected tuberculous lesioned lymph node with haemotoxylin-eosin (H&E) staining, X40 observation. A. lymphocytes, plasma cells, and monocytes. B. central caseous necrosis

Histopathologically, granulomatous changes were observed with tissue cells and central caseous necrosis only from the two AFB smear-positive tuberculous lesions, (Table 2). Focal necrosis, dystrophic calcification, and foreign body-type giant cells were also seen in both of the two AFB smear-positive tuberculous lesioned samples, (Figure 4).

## Discussion

Given the future possible intensification of cattle production and the subsequent increase in the likelihood of zoonotic transmission of BTB in Ethiopia [46], such a prevalence estimate was considered important. In our study, an overall prevalence of 9.1% BTB was found among slaughtered cattle based on a detailed postmortem examination. In Ethiopia, there have been different reports of BTB prevalence using abattoir-based studies in different areas [38,47], which is comparable with our findings. However, the overall prevalence of BTB in this study was lower than that reported by Ameni *et al*. [48] in Wolayta Sodo and Elias *et al*. [49] in Addis Ababa abattoirs, Ethiopia, but higher than the other reports in the country [50,51]. These prevalence report variations might be due to the sample size considered by various studies and/or the difference in the management and production systems of the slaughtered cattle. In our study area, while intensive and commercial farming is less practiced, BTB prevalence may be lower than that of the other areas of the country. In addition, the prevalence reports of BTB among studies might be explained by differences in the interactions between the livestock and wildlife across varying study regions and to the widest host range that *M. bovis* can infect [52]. Several studies have also indicated that not all cattle infected with *M. bovis* have gross tuberculous lesions that are visible in the tissues examined at slaughter [53]. The lower sensitivity and specificity of gross postmortem inspection might also influence the method employed for reporting positive cases and the anatomical sites considered [43,54–56], implicating that some animals with tuberculous lesions might be missed by the postmortem method.

A study in Ethiopia indicated that BTB is one of the major causes of carcass condemnation due to TB and TB-like lesions [57], as also shown in our findings by detailed meat inspection procedures including frequent slicing of organs and lymph nodes. However, some studies exhibited low sensitivity of routine abattoir inspection; detected only 15.15% out of the carcasses with visible lesions, implying 84.5% missed opportunities [50]. Therefore, given the frequently practiced routine abattoir inspection, there might be lots of missed lesions; which could contribute to the distribution of unsafe meat for the public. In contrast to the report by Dechassa [2014, 50], a study conducted in Ghana recommended that if done proficiently, visual inspection at necropsy could serve as the primary screening measure for beef contaminated with mycobacterial species in abattoirs in resource-poor settings [58]. Differences among the diverse studies, including the current study might be explained by the different examination procedures followed, routine abattoir inspections, or detailed postmortem examination. Therefore, particular attention should be given to appropriate surveillance of BTB in slaughtered cattle for the success of the TB control program in livestock thereby in the public.

In our findings, we observed only 4.4% of AFB positive samples among the total tuberculous lesion using bacteriological (ZN staining) and histopathological methods. As shown by the previous report [59] ZN can detect more AFB positive samples as tuberculous bacilli that might be present in the lesions obtained from slaughtered cattle. This agreed with the previous prevalence reports [59–61] using the bacteriological method. Different studies have also reported a varied AFB positivity ranging from 0% to as high as 75% [62]. In our study, the decontamination process might minimize our ability to accurately detect AFB from the collected BTB suspected tuberculous lesions. Histologically, granulomatous changes were observed only in the lymph nodes that had gross lesions [12]. In our study, the lesions were typical for BTB with granulomas that had central caseous necrosis and calcification and confirmed also the acid-fast organisms by ZN that were present in these lesions.

In the present study, BTB suggestive lesions were found distributed across lymph nodes of the lungs, in which the majority were found on mediastinal followed by bronchial lymph nodes. This finding was in line with previous studies [16,38,63] who reported that the majority of TB suggestive lesions were distributed across the lymph nodes of lungs among cattle slaughtered at Bahir Dar abattoir, Ethiopia. These agreeable results more likely indicate that most of the BTB infection is acquired by aerosol transmission route thus inhalation might be the principal route of infection in cattle [52]. In addition, there was a difference in the frequency of tuberculous lesions in the animal body conditions. A significantly higher frequency of tuberculous lesions was documented from cattle with poor body conditions. These findings were consistent with the results recorded in many of the previous reports in different corners of the country including in Dilla [60], Adama [64], Gondar [65], and Hosanna [54], and Butajira [49]. This may indicate that cattle with poor body condition might have a lower protective mechanism against invading agents and thus, either they are immunocompromised due to concurrent infections or malnourished [66]. Possible risk factors including management practices, and herd size might contribute to the association of BTB tuberculous lesions and poor body conditions [52,67].

## Conclusion

The findings from this study have documented the prevalence of BTB in cattle in North Gondar, Ethiopia using data from postmortem inspections at the abattoir. A higher proportion of tuberculous lesions were identified in the lymph nodes of the lungs; the mediastinal and bronchial lymph nodes and followed by the mesenteric lymph nodes and lymph nodes of the head. Thus, the findings highlight the importance of continuous abattoir investigations of BTB in animal populations and need feasible and practical control strategy of the disease in the region.
